# Pharmacokinetic Study of Bioactive Glycopeptide from *Strongylocentrotus droebachiensis* After Intranasal Administration to Rats Using Biomarker Approach

**DOI:** 10.3390/md17100577

**Published:** 2019-10-11

**Authors:** Alexander N. Shikov, Olga N. Pozharitskaya, Natalia M. Faustova, Vera M. Kosman, Valery G. Makarov, Ebrahim Razzazi-Fazeli, Johannes Novak

**Affiliations:** 1 St. Petersburg State Chemical Pharmaceutical University, Prof. Popov, 14, 197376 Saint-Petersburg, Russia; 2 St. Petersburg Institute of Pharmacy, Leningrad Region, Vsevolozhsky District, Kuzmolovo P 245, 188663 Saint-Petersburg, Russia; olgapozhar@mail.ru (O.N.P.); faustova-78@mail.ru (N.M.F.); kosmanvm@mail.ru (V.M.K.);; 3 Vetcore facility for Research, University of Veterinary Medicine, Veterinärplatz 1. 1210 Wien, Austria; Ebrahim.Razzazi@vetmeduni.ac.at; 4 Institute of Animal Nutrition and Functional Plant Compounds, University of Veterinary Medicine, Veterinärplatz 1. 1210 Wien, Austria; Johannes.Novak@vetmeduni.ac.at

**Keywords:** *Strongylocentrotus droebachiensis*, rats, plasma, nose mucosa, liver, kidney, spleen, striated muscle

## Abstract

A glycopeptide fraction (GPF) from internal organs of green sea urchins (*Strongylocentrotus droebachiensis* Müller, Strongylocentrotidae) has been reported to be an effective bronchitis treatment. In this study, we evaluated the pharmacokinetic and tissue distribution of GPF, following single and repeated intranasal (i/n) administration over the course of seven days in rats. The method measuring lactate dehydrogenase as biomarker was used to analyse the plasma and tissue concentrations of GPF. GPF appears in the plasma 15 min after single i/n administration (100 µg/kg) and reaches its maximum at 45 min. The area under the curve (AUC)_0–24_ and C_max_ were similar using both i/n and intravenous administration, while mean residence time (MRT) and T_1/2_ after i/n administration were significantly higher compared with intravenous (i/v) administration. The absolute bioavailability of GPF after i/n administration was 89%. The values of tissue availability (f_t_) provided evidence about the highest concentration of GPF in the nose mucosa (ft = 34.9), followed by spleen (ft = 4.1), adrenal glands (ft = 3.8), striated muscle (ft = 1.8), kidneys (ft = 0.5), and liver (ft = 0.3). After repeated dose administration, GPF exhibited significantly higher AUC_0–24_ and MRT, indicating its accumulation in the plasma.

## 1. Introduction

Recently, marine organisms have become a promising source of therapeutic agents based on their broad panel of bioactivities. Sea urchins are renewable marine species, which are commercially harvested and processed in the food industry and cosmetics, and have a great potential for the development of medicines. All parts of these unique organisms including the body wall, gonads, coelomic fluid, and internal organs show a high medicinal value [1,2,3,4,5,6,7,8]. 

Green sea urchins *Strongylocentrotus droebachiensis* Müller (Strongylocentrotidae) belong to an abundant group of marine organisms, which colonize different regions of the Atlantic Ocean including the Barents Sea [9]. Antiallergic, radical scavenging, and hypoglycaemic effects are reported for pigments from *S. droebachiensis* [7]. Lipids from the body walls and gonads of green sea urchins showed anti-inflammatory [10] and antidiabetic activities [11]. Coelomites of *S. droebachiensis* were active against *Vibrio anguillarum, Escherichia coli*, and *Corynebacterium glutamicum* [12]. Two antineoplastic glycoproteins (strongylostatin 1 and 2) were isolated from the whole body of *S. droebachiensis* [8]. Cysteine-rich antimicrobial peptides strongylocins 1 and 2 with molecular weights of 5.6 and 5.8 kDa, respectively, and dimeric centrocins 1 and 2 with molecular weights of 4.5 and 4.4 kDa, respectively, were purified from coelomites of green sea urchins [13,14]. SpStrongylocins, homologues of strongylocins were isolated from sea urchins *S. purpuratus* [15]. Recently, Solstad et al. [16] reported new antimicrobial peptides EeCentrocin 1 and 2 and EeStrongylocin 2 from *Echinus esculentus*. These compounds contain a cationic heavy chain of 30 and 32 amino acids and a light chain of 13 amino acids, respectively. The synthetically derived centrocin 1 heavy chain peptide and its derivatives comprising 30 amino acids showed anti-inflammatory activity in vitro. All these peptides markedly reduced the release of the pro-inflammatory cytokine Tumor Necrosis Factor-α (TNF-α) in LPS-stimulated macrophages derived from the human monocytic cell line THP-1 [17]. The brominated heavy chain unit of centrocin 1 (CEN1HC-Br) downregulates interleukin (IL)-12p40, IL-6, IL-1β, TNF-α, and Toll-Like Receptor 2 (TLR2) expression in the model of rat ear swelling induced by *Propionibacterium acnes* [18].

Recently, we isolated a glycopeptide fraction (GPF) from internal organs of *S. droebachiensis*. The GPF showed potent anti-inflammatory effects, especially for the treatment of bronchitis. In-vitro, GPF inhibited Cyclooxygenase2 (COX2), LPS-induced p38 Mitogen-Activated Protein Kinase (MAPK) phosphorylation by blocking TLR4 [19]. In vivo, GPF was active in models of acute and chronic bronchitis induced by tobacco smoking and formalin after intranasal (i/n) administration [20,21]. 

Historically, the focus in the development of natural products was set on efficiency and specificity, while pharmacokinetics was often taken for granted because most natural products were “designed” by natural selection to avoid metabolic degradation and to cross biomembranes [22]. One of the major determinants of success or failure for new medicines is its pharmacokinetic properties. A direct analysis of active compounds [23,24,25] or its metabolites [26,27] has been reported for the study of pharmacokinetics of marine derived small molecules in vivo. General principles of pharmacokinetics are applicable also for animal-derived medicines. However, direct analysis of high molecular weight compounds derived from animals poses extra challenges because of structural complexity, similarity to endogenous molecules, and lack of specificity and sensitivity of bioanalytical assays. An approach using biomarkers is recommended for pharmacokinetic study of such compounds [28]. 

We have not found pharmacokinetic and tissue distribution studies of marine-derived glycopeptides in the literature. Therefore, we decided to investigate the pharmacokinetics and tissue distribution of GPF from internal organs of *S. droebachiensis* after single and multiple intranasal administrations to rats using the biomarkers approach. 

## 2. Results

### 2.1. Characterisation of GPF

A shotgun proteomic approach was carried out to charaterize the proteins. The database search revealed 333 proteins with 1353 peptides. A complete list of the identified proteins and the appropriate chromatogram can be found in the supplementary data (Appendix A). All proteins with at least 20 peptides are shown in Table 1.

The sugar composition was analysed by high-performance liquid chromatography−refractive index detection (HPLC-RID) after trifluoroacetic acid derivatization. The main monosacharides in GPF were fucose (15.4 ± 0.1 mg/g) and glucose (86.2 ± 0.5 mg/g).

### 2.2. Method Validation

**Figure 1:**
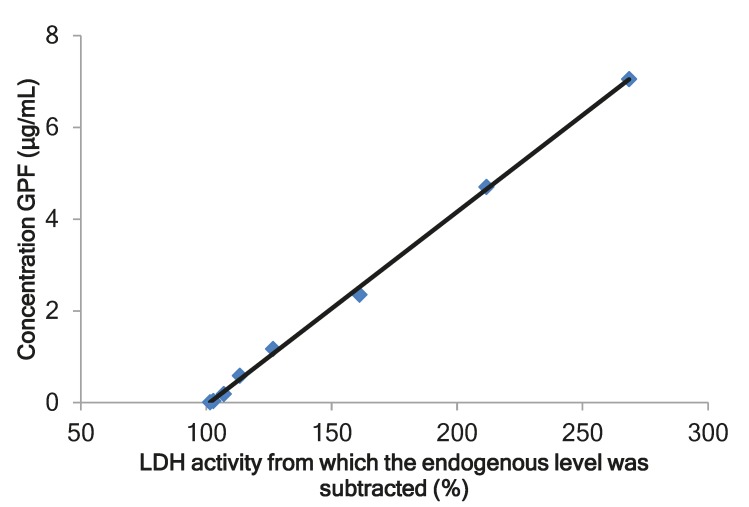
The calibration curve for the calculation of glycopeptide fraction (GPF) in plasma. LDH, lactate dehydrogenase.

The biomarkers approach is recommended by the Food and Drug Administration (FDA) for pharmacokinetic study of complex substances derived from animals [28]. Our previous experiments in rats showed a dose-dependent decrease of the number of pro-inflammatory cytokines and leucocytes in bronchoalveolar lavage (BAL) after intranasal administration of GPF to rats with bronchitis [20,21]. A direct correlation was observed between the level of lactate dehydrogenase (LDH) and its isoenzymes in pleural fluid and in BAL in the case of lung tissue damage and pulmonary endothelial cell injury [29]. On the basis of these data, LDH was selected as biomarker. A comparison of the data of the determination of LDH activity of intact plasma with calibration samples of GPF showed a dose-dependent change. The calibration curve for GPF was linear over a concentration range of 0.01–7.05 μg/mL (Figure 1). The GPF concentration was calculated according to the following equation: *у =* 0.042*x* − 4.274 (r = 0.9995), where *у* is the concentration of GPF (μg/mL) and *х* is the LDH activity from which the endogenous level was subtracted (%). The validation data for the method of determining GPF concentration in plasma are presented in Table 2.

To verify the selectivity of the analytical method, an analysis of the intact biomaterial (tissues/organs) and model samples of liver, kidney, and nose mucosa homogenates with the addition of GPF was performed. The data showed that GPF affects the concentration of the enzyme LDH in the homogenates in a dose-dependent manner. The validation data for the method for determining of GPF concentration in tissues/organs are presented in Table 3.

### 2.3. Pharmacokinetic and Tissue Distribution

GPF was well tolerated: no clinical signs of toxicity as changes in locomotor activity, touch response, aggression, tremor, convulsions, pain, or mortality were observed in rats after intravenous (i/v) (100 µg/kg) as well as after i/n (50, 100 and 200 µg/kg) administration of GPF. No toxic effects were observed in rats after seven days of repeated i/n administration of GPF (3 × 100 µg/kg a day) as well.

**Figure 2:**
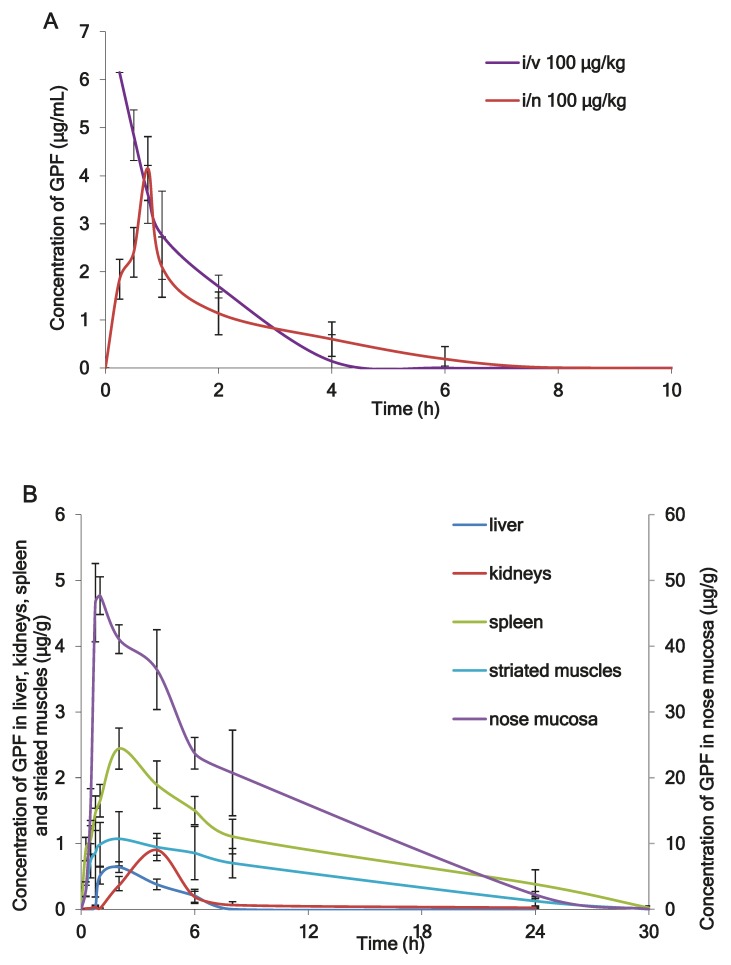
(**A**) The mean plasma profiles of GPF after intravenous (i/v) administration and intranasal (i/n) administration in dose 100 µg/kg to the rats (*n* = 5), (**B**) The mean tissues profiles of GPF after i/n administration (100 µg/kg) to the rats (*n* = 5).

**Figure 3:**
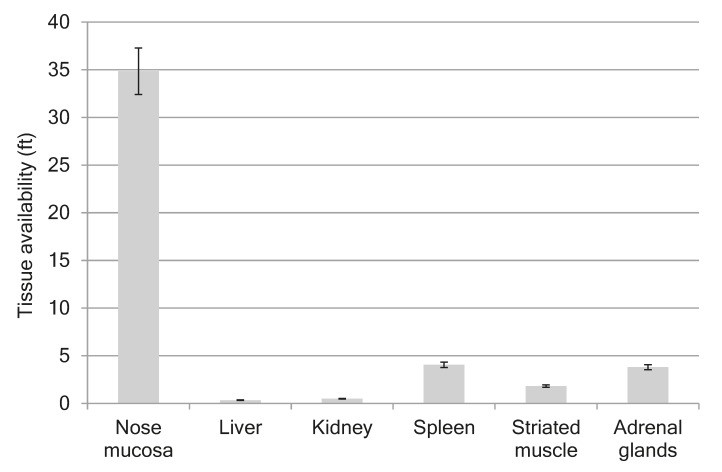
Tissue availability of GPF after i/n administration in dose 100 µg/kg to the rats.

Figure 2A shows the mean plasma profiles of GPF after i/v and i/n administration to the rats at the dose of 100 µg/kg. The mean profiles of GPF in liver, kidneys, spleen, striated muscle, and nose mucosa after i/n administration (100 µg/kg) to the rats are presented in Figure 2B, while the tissue availability of GPF after i/n administration is presented in Figure 3. The pharmacokinetic parameters of GPF distribution in plasma, nose mucosa, liver, kidneys, spleen, striated muscle, and adrenal glands are presented in Table 4. 

**Figure 4:**
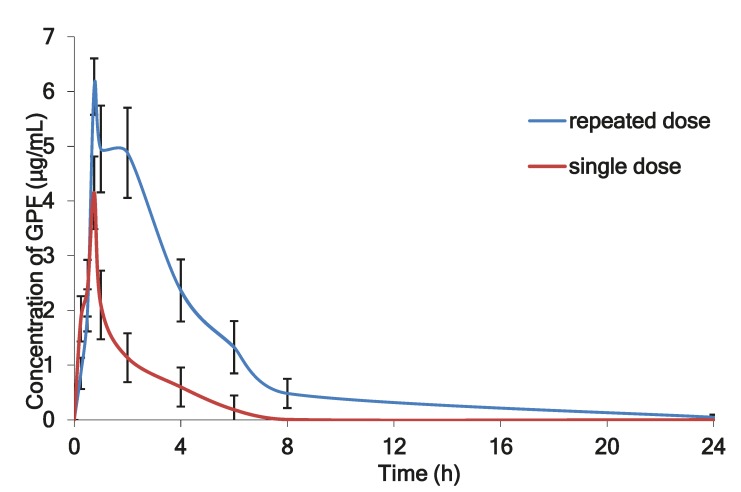
The mean plasma profiles of GPF after i/n administration to the rats after single (100 µg/kg) and repeated doses (3 × 100 µg/kg during seven consecutive days).

Figure 4 shows the mean plasma profiles of GPF after single and repeated doses of i/n administration to the rats.

## 3. Discussion

The composition of animal-derived medicinal products is very complicated [30]. Just a few peptides have been reported from sea urchins, and limited information is available about its structure [8,13,14,16,18]. Using tandem mass spectrometry LC-MS/MS, we successfully identified the number of proteins and peptides in GPF (Table 1, Appendix A), while HPLC-RID led to identification of two monosaccharides: fucose and glucose (Appendix A). 

For pharmacokinetic study of such complex substances derived from animals, the FDA recommends a direct measurement of the pharmacological effect of the drug using the biomarkers approach [28]. Immunoassays and bioassays are among the most useful techniques for this [31]. The bioassay was defended as “an analytical procedure measuring a biological activity of a test substance based on a specific, functional, and biological response of a test system” [32]. An in vivo bioassay includes the administration of the tested substance to animals followed by the measurement of the response in the organism [33]. The selection of response biomarker is very important for a bioassay. The biomarkers approach has been widely used for pharmacokinetic studies of high molecular weight compounds in vivo and in volunteers [33,34,35,36].

Biomarkers should reflect drug action. Previously, we observed that the thickness of bronchial tissue and the number of leucocytes in BAL dose-dependently decreased after inhalation administration of GPF to rats in the model of bronchitis [20]. The number of pro-inflammatory cytokines in BAL was decreased in rats with modulated acute bronchitis after inhalation administration of GPF [21]. The direct correlation of LDH and its isoenzymes in pleural fluid, as well as in BAL, was suggested as one of the biomarkers of lung tissue damage and pulmonary endothelial cell injury [29]. 

Our previous experiment shows correlation between the level of LDH and GPF concentration in vitro. Therefore, we considered to use LDH as biomarker and to establish a bioassay method for detecting GPF based on its activity to LDH in rats. The method was developed, validated, and successfully applied for pharmacokinetic studies of GPF after single and multiple i/n administrations to rats.

Concentrations of GPF in plasma and tissues and the corresponding times were taken directly from the raw data as a mean of five rats (Figure 2A,B), while pharmacokinetic parameters of GPF were calculated from the concentration-time data using a noncompartmental pharmacokinetic model (Table 4). After i/v administration of GPF (100 µg/kg), the mean plasma concentration–time curve declined in a polyexponential manner (Figure 2A) with T_1/2_ of 0.8 h and mean residence time of 1.11 h (Table 4). GPF appears in plasma 15 min after single i/n administration (100 µg/kg) and reaches a maximum at 45 min (Figure 2A). The AUC_0–24_ and C_max_ were similar using both routes of administration, while MRT and T_1/2_ after i/n administration were significantly higher compared with i/v administration (Table 4). The absolute bioavailability of GPF after i/n administration was 89%. This fact confirms the rationality of the intranasal route of administration of GPF. 

The highest concentration of GPF after i/n administration (C_max_ = 53.661 µg/g) was found in the nose mucosa, while the lowest level was found in the liver (C_max_ = 0.73 µg/g) (Table 4). The values of tissue availability (f_t_) provided evidence of the highest concentration of GPF in the nose mucosa (ft = 34.9), followed by spleen (ft = 4.1), adrenal glands (ft = 3.8), and striated muscle (ft = 1.8). Minimal concentrations of GPF were measured for liver (ft = 0.3) and kidneys (ft = 0.5) (Figure 3). 

It was found that pharmacokinetics of GPF given by nasal route were linear in the range of doses of 50–200 µg/kg, AUC_0-24_, T_1/2_, and С_max_ were evidently increased after dose increasing, but wide data variations were observed (Table 4). Because, in the experiment with a single administration of GPF, the linear pharmacokinetic was established, one dose level (100 μg/kg) was used in experiments with multiple dose administration.

Figure 4 illustrates the mean plasma concentration of GPF versus time profile after single dose (100 µg/kg) and after repeated daily dosing with 3 × 100 µg/kg of GPF during seven days. After repeated dose administration, GPF exhibited significantly higher AUC_0–24_ (22.98 μg·h/mL) and long circulation time (60.04 h), which evidenced about its accumulation in the plasma after repeated administration (Table 4). 

We believe that the results of this study confirm the usefulness of the biomarkers approach for the study of pharmacokinetics of marine-derived complex substance mixtures. The developed and validated method showed good accuracy, precision, and sensitivity. 

Marine-derived peptides and glucans showed potent anti-inflammatory activity and can used for the treatment of respiratory viral infections and their complications [37]. Because of their susceptibility to enzymatic degradation and other specificity, the administration of peptides is mostly limited to invasive injections, which can be painful and inconvenient. Over the past decades, the nasal route has gained much attention as a noninvasive alternative for systemic delivery of various classes of peptide drugs [38]. The high surface area available for absorption, the highly vascularized layer of the mucous membrane, and the fact that the nasal cavity appears to have very little metabolizing ability suggest that absorption through the nasal membranes is the optimal delivery method for peptides. However, a possible nasal hypersecretion as reaction to the application may become a limitation for the intranasal administration route. However, our results are consistent with published data suggesting the expanding use of intranasal delivery of therapeutic peptides as an important treatment strategy for many diseases. Nose-to-brain delivery offers to peptide drugs the possibility to reach the brain in a non-invasive way [39]. Intranasal insulin rapidly improves hepatic energy metabolism, and reduces the hepatic fat in humans [40]. Recently, Huang et al. summarized the neuroprotective effects of intranasal administration peptides against cerebral ischemic stroke [41]. It was shown that nasal delivery of large peptides such as parathyroid 1-34 can benefit from a permeation enhancer to promote absorption across the nasal mucosa into the bloodstream [42]. We believe that the nasal route of administration of GPF is a very attractive alternative to injections because of its convenience, which should assure a good compliance by patients.

## 4. Materials and Methods

### 4.1. Materials

Green sea urchins *Strongylocentrotus droebachiensis* were harvested in Barents Sea in August 2016. The organisms were identified by Dr. Irina Urakova, and a voucher specimen (SDBS12) was deposited in St. Petersburg Institute of Pharmacy (St. Petersburg, Russia). Analytical grade chemicals and solvents for extraction and assay were purchased from local chemical suppliers. 

### 4.2. Extraction Procedures

Sea urchins were dissected, gonads were removed, coelomic fluid with the rest of internal organs was collected, and five volumes of purified water were added. The mixture was macerated for 15 min with constant stirring at 95 °C, and for 2 h at room temperature. After cooling in the refrigerator, the mixture was centrifuged at 3000 rpm for 15 min. The resulting supernatant was concentrated under vacuum at 40 °C, and ethanol (95%) was added. After precipitation of high molecular weight proteins, the solution was filtered and concentrated under vacuum. The concentrate was pooled with ethanol (95%) and, after stirring, was cooled in the refrigerator for 16 hours. The mixture was centrifuged at 3000 rpm for 15 min; supernatant was concentrated under vacuum, filtered, and dialyzed against purified water; and the protein recovered by lyophilisation.

### 4.3. LC-MS/MS Analysis

For the protein/peptide identification, 10 mg of the lyophilized GPF was resolubilized in 100 µL PBS. Protein concentration was determined using Pierce 660nm reagent (Thermo Fisher Scientific, Waltham, MS, USA) using a NanoDrop spectrophotometer (Thermo Fisher Scientific, Waltham, MS, USA). Then, 30 µg of the protein was filled up to 150 µL with 8 M Urea (Roth, Karlsruhe, Germany) in 50 mM TRIS (Sigma, St. Louis, MI, USA). Reduction and alkylation were performed in a reaction tube before samples were applied onto the filter. Reduction was achieved with 200 mM dithiothreitol (DTT, Roth, Karlsruhe, Germany) to obtain a concentration of 20 mM DTT on a thermomixer (30 min, 37 °C). This was followed by alkylation with 500 mM iodacetamide (IAA, Sigma, St. Louis, MI, USA) in a final concentration of 60 mM IAA performed for 30 min at room temperature. Reduced and alkylated proteins and peptides were loaded onto the filter (Amicon 10 kDa, Sigma, St. Louis, MI, USA). The solution was centrifuged for 20 min at 10000 rcf. The flow-through was collected as it contains peptides with a size of up to around 10 kDa. Before LC-MS analysis, peptide extracts were desalted and cleaned up using C18 spin tips (Pierce, Waltham, MS, USA) according to the manufacturer’s protocol.

The flow-through peptide sample was dissolved in 15 µL 0.1% trifluoroacetic acid (TFA, Fisher Scientific, Waltham, MS, USA) and 6 µL was injected into the LC-MS system. Peptides were separated on a nano-HPLC Ultimate 3000 RSLC system (Thermo Fisher Scientific, Waltham, MS, USA). Sample pre-concentration and desalting was accomplished with a 5 mm Acclaim PepMap μ-Precolumn (300 µm inner diameter, 5 µm particle size, and 100 Å pore size) (Thermo Fisher Scientific, Waltham, MS, USA). For sample loading and desalting, 2% acetonitrile (ACN, Merck, Darmstadt, Germany) in ultra-pure H_2_O with 0.05% TFA was used as a mobile phase with a flow rate of 5 µL/min. Separation of peptides was performed on a 25 cm Acclaim PepMap C18 column (75 µm inner diameter, 3 µm particle size, and 100 Å pore size, Thermo Fisher Scientific, Waltham, MS, USA) with a flow rate of 300 nl/min. The gradient started with 4% B (80% ACN with 0.1% formic acid) and increased to 35% B in 60 min. This was followed by a washing step with 90% B. Mobile phase A consisted of ultra-pure H_2_O with 0.1% formic acid. 

For mass spectrometric analysis, the LC was directly coupled to a high resolution Q Exactive HF Orbitrap mass spectrometer (Thermo Fisher Scientific, Waltham, MS, USA). MS full scans were performed in the ultrahigh-field Orbitrap mass analyzer in ranges of m/z 350−2000 with a resolution of 60,000, maximum injection time (MIT) of 50 ms, and automatic gain control (AGC) set to 3e^6. The top 10 intense ions were subjected to Orbitrap for further fragmentation via high energy collision dissociation (HCD) activation over a mass range between m/z 200 and 2000 at a resolution of 15,000 with the intensity threshold at 4e^3. Ions with charge state +1, +7, +8, and >+8 were excluded. Normalized collision energy (NCE) was set at 28. For each scan, the AGC was set at 5e^4 and the MIT was 50 ms. Dynamic exclusion of precursor ion masses over a time window of 30s was used to suppress repeated peak fragmentation.

Spectra were searched in UniProt/Trembl (downloaded from the publicly available servers (http://www.uniprot.org) with the taxonomy “Strongylocentrotus tx[7664]” containing 112 reviewed and 30,190 unreviewed protein entries, as well as a crap database containing common contaminations (https://www.thegpm.org/crap/).

As the sample was not digested with trypsin or any other protease, the database search was performed with “no enzyme” in Proteome Discoverer 2.2 (Thermo Fisher Scientific, Waltham, MS, USA). Search parameters were set to carbamidomethylation on cysteins as fixed modification, deamidation on asparagine and glutamine, oxidation on methionine, and C-terminal amidation as dynamic modifications. Precursor mass tolerance was set to 10 ppm, fragment mass tolerance to 0.02 Da, and two missed cleavages were allowed. The false discovery rate (FDR) was set to 1%. Uncharacterized proteins were blasted (https://www.uniprot.org/blast/) to obtain common protein names.

### 4.4. Monosaccharide Analysis

For the analysis of the monosaccharides, GPF was treated with trifluoroacetic acid. Hydrolysate was analysed by high-performance liquid chromatography (Shimadzu HPLC system, Kyoto, Japan) according to a method [43]. The separation of monosaccharides was performed on a Asahipak NH2P-50E 4.6 × 250 mm (Shodex, Japan) column with acetonitrile as mobile phase.

### 4.5. Animals

Male outbred rats were obtained from Rapplovo breeding house (St. Petersburg, Russia). Rats (*n* = 5 per time point) were fasted overnight before the experiment. The animals were kept under standard conditions with a 12 h light–dark cycle, at ambient temperature (22 ± 2 °C), and relative humidity of 60% ± 10%. The animals had free access to a standard laboratory diet (standard diet: Tosno, Russia) and water ad libitum. Rats were divided into five groups: group A, intravenous (i/v) injection of GPF (single dose, 100 µg/kg) for determination absolute bioavailability; groups B, C, and D, intranasal (i/n) administration of GPF (single dose of 50, 100, and 200 µg/kg, respectively); and group E, intranasal (i/n) administration of GPF (100 µg/kg, three times a day) during seven days. It was shown that GPF was not toxic in rats after chronic endotracheal administration at the dose of 0.2 mg/kg, and it was not toxic after acute intraperitoneal injection to rats at the dose of 5.0 mg/kg [44]. The significant anti-inflammatory effects of GPF were observed in rats at doses of 50 and 100 µg/kg [20].

An aqueous solution of GPF (1 mg/mL) was used for the administration to rats. After administration, the rats were euthanized in a CO_2_ chamber at the time points of 0, 0.25, 0.5, 0.75, 1, 2, 4, 6, 8, and 24 h. The animals from the group E were euthanized on the seventh day of experiment. The blood was collected in heparine tubes by cardiac puncture, centrifuged at 2000× *g* for 15 min at 4 °C, and plasma was collected and stored at −20 °C. The tissues/organs with different vascularity (spleen, adrenal glands, nose mucosa, tissue muscle, liver, and kidneys) were removed by surgical resection. Each organ sample was weighed and thoroughly homogenized in 0.1 M Tris-HCl buffer (pH 8.0). The nasal mucosa was washed twice with 0.1 M Tris-HCl buffer and treated in the ultrasound bath at 37 °C for 30 min with the same buffer. After vortex-mixing and centrifugation for 15 min at 6000× *g*, the upper phase was collected and used for LDH assay.

The experiments were approved by the Ethical Commission of the St. Petersburg Institute of Pharmacy (Leningrad Reg., Vsevolozhsky Distr., Kuzmolovo P 245, Russia), (protocol # 1.17/17 dated on 29.03.2017), and were performed according to the EEC Directive of 1986 (86/609/EEC).

### 4.6. Analysis of LDH Activity

The LDH activity was determined photometrically (A25, Biosystems S.A., Spain) using a Lactate Dehydrogenase Activity Assay Kit (Biosystems S.A., Spain). LDH catalyzes the reduction of pyruvate by reduced Nicotinamide Adenine Dinucleotide (NADH), to form lactate and NAD^+^. The catalytic concentration is determined from the rate of decrease of NADH, measured at 340 nm. The test solutions were prepared by mixing of plasma or calibration solutions of GPF or a calibrator solution with an appropriate volume of 0.1 M Tris-HCl buffer (pH 8.0) to fit with concentration in a linear range, while organ homogenate samples were used without dilution. The appropriate dilution coefficient was used for calculations. The endogenous level of products reacted with the LDH activity kit was subtracted at each time point in each sample. The appearance of lactate, determined photometrically, is proportional to the concentration of GPF in a plasma/organ sample. The results were initially expressed as total LDH activity (µmol units/L) per sample, and the concentration of GPF in samples was calculated in µg/mL for plasma or µg/g for organ/tissue. The International Conference on Harmonization (ICH) guidelines on the validation of analytical methods were used [45,46].

### 4.7. Pharmacokinetic and Statistical Analysis

A PKSolver add-in for the Excel was used for the pharmacokinetic calculations for GPF in organs/tissues and plasma. The parameters were calculated from the concentration-time data using a noncompartmental pharmacokinetic model, as described previously [36]. The results are expressed as the mean ± standard deviation (*n* = 5 for each time point).

## 5. Conclusions

We evaluated for the first time the absorption and pharmacokinetics of GPF following single and repeated intranasal administration over the course of seven days in rats. The biomarkers approach using correlation between the level of LDH and GPF concentration was used. Our results show that GPF is rather well absorbed from the nasal mucosa. No adverse effects were noted after i/v and single and repeated i/n administration routes in this study. The concentration versus time profile for i/n GPF suggests it may provide an effective, non-invasive delivery route. The results of pharmacokinetic studies may help clinicians to optimise the mode of administration of GPF in clinics.

## Figures and Tables

**Table 1:** Identified proteins with at least 20 peptides per protein.

Protein Name (Accession Number)	SC [%]	# Peptides	MW [kDa]
Major yolk protein (P19615)	39	132	153.9
* Filamin-B (W4YPA7)	19	79	279.1
* Transforming growth factor-beta-induced protein ig-h3 (W4Y3B7)	53	65	38.5
* Ubiquitin-40S ribosomal protein S27a (W4XMU0)	49	30	17.8
Actin, cytoskeletal 1A (A0A0C3SG04)	51	40	41.8
* Apolipoprotein B-100 (W4ZKG2)	5	43	614.6
Actin, muscle OS = Strongylocentrotus purpuratus (W4XTP5)	40	37	41.7
Major yolk protein (Fragment) (A0A1B4XK6)	42	32	44.8
* Apolipoprotein B-100 (W4ZKG3)	9	29	202.8
* Myosin heavy chain, striated muscle (W4Y2X0)	4	23	225.1
* Transforming growth factor-beta-induced protein ig-h3 (W4XSF0)	37	21	37.7

* Blast results; SC [%]: sequence coverage; # Peptides: number of identified peptides, MW [kDa]: molecular weight.

**Table 2:** The validation data for the method of determining the glycopeptide fraction (GPF) in plasma.

Parameter	Range
Accuracy, *%*	
ULOQ (7.05 μg/mL)	1.7–1.8
Middle-quality control (2.35 μg/mL)	3.1–11.1
Low-quality control (0.024 μg/mL)	0.3–13.6
LLOQ (0.01 μg/mL)	3.3–13.4
Intraday//Interday precision (RSD), *%*	
ULOQ (7.05 μg/mL)	5.0//3.7
Middle-quality control (2.35 μg/mL)	6.6//2.6
Low-quality control (0.024 μg/mL)	2.8//1.6
LLOQ (0.01 μg/mL)	0.7//1.6

ULOQ, upper limit of quantification; LLOQ, lower limit of quantification; RSD, relative standard deviation.

**Table 3:** The validation data for the method of determining GPF in tissues/organs.

Parameter	Kidney	Liver	Nose Mucosa
Linearity (μg/mL homogenates)	0.007–2.64	0.005–0.076	0.1–51.6
Regression equation	*y* = −72.45·lg*x* + 24.73	*y* = 27.25 *x* + 72.01	*y* = 61.59 *x* + 110.97
Correlation coefficient r	0.9993	0.9951	0.9958
Accuracy (kidney/liver/nose mucosa), *%*			
ULOQ (1.60/0.076/4.7 μg/mL)	2.9–10.1	1.0–8.2	1.3–3.1
Middle-quality control (0.53/0.035/2.35 μg/mL)	5.8–11.6	3.8–11.8	1.0–3.2
Low-quality control (0.013/0.014/0.19 μg/mL)	0.7–14.7	2.8–11.7	4.6–12.3
LLOQ (0.004/0.005/0.06 μg/mL)	1.5–18.5	4.7–17.8	3.5–8.5
Intraday//Interday precision (RSD), *%* (kidney/liver/nose mucosa)			
ULOQ (1.60/0.076/4.7 μg/mL)	1.4//3.5	2.2//4.2	1.7//5.1
Middle-quality control (0.53/0.035/2.35 μg/mL)	1.4//5.6	0.7//5.8	0.7//4.2
Low-quality control (0.013/0.014/0.19 μg/mL)	0.9//6.8	2.3//6.7	2.4//10.3
LLOQ (0.004/0.005/0.06 μg/mL)	2.9//10.9	2.9//9.8	2.4//7.5
LOD (μg/mL homogenates)	0.007	0.005	0.1

ULOQ, upper limit of quantification; LLOQ, lower limit of quantification; LOD, limit of detection; RSD, relative standard deviation.

**Table 4:** Pharmacokinetic parameters of GPF after single dose 100 µg/kg and repeated dose administration to rats.

Sample	Dose	Parameter				
	(µg/kg)	AUC_0–24_ (µg·h/g)	MRT (h)	T_1/2_ (h)	C_max_ (μg/g)	T_max_ (h)
Single dose						
Plasma *, i/v	100	8.00 ± 1.73	1.11 ± 0.26	0.80 ± 0.17	6.15 ± 1.29	-
Plasma *, i/n	50	3.93 ± 1.78	1.54 ± 0.35	0.77 ± 0.33	2.90 ± 0.85	0.67 ± 0.20
Plasma *, i/n	100	7.14 ± 5.50	5.58 ± 5.50	3.53 ± 3.27	4.15 ± 1.63	0.75 ± 0.05
Plasma *, i/n	200	12.64 ± 5.98	4.62 ± 4.61	4.03 ± 3.89	6.22 ± 1.51	0.70 ± 0.10
Nose mucosa, i/n	100	248.75 ± 24.45	8.00 ± 4.53	4.46 ± 3.03	53.66 ± 8.01	0.85 ± 0.14
Liver, i/n	100	2.40 ± 0.71	7.40 ± 7.46	4.48 ± 5.26	0.73 ± 0.20	1.60 ± 0.55
Kidneys, i/n	100	3.50 ± 1.85	8.07 ± 3.38	6.42 ± 2.91	0.98 ± 0.37	3.60 ± 0.89
Spleen, i/n	100	28.90 ± 7.24	10.20 ± 2.88	6.48 ± 2.09	2.53 ± 0.70	2.40 ± 0.89
Striated muscle, i/n	100	12.98 ± 9.05	8.18 ± 5.42	4.98 ± 3.38	1.74 ± 1.28	1.85 ± 2.33
Adrenal glands, i/n	100	27.06 ± 6.73	21.42 ± 8.30	14.69 ± 5.89	2.67 ± 1.17	2.40 ± 0.89
Repeated dose						
Plasma *, i/n	3*100	22.98 ± 12.68	60.04 ± 21.90	3.70 ± 1.63	6.99 ± 1.32	1.00 ± 0.50

* AUC_0–24_ (μg·h/mL) for plasma; C_max_ (μg/mL) for plasma; AUC_0–24_, the area under the curve; MRT, mean residence time; T_1/2_, apparent half-life of elimination. The results are expressed as the mean ± SD (*n* = 5).

## Appendix A

The complete list of the identified proteins and the appropriate chromatogram, HPLC-RID chromatograms for reference sugars and identified sugars.
